# Interactions of HMGB Proteins with the Genome and the Impact on Disease

**DOI:** 10.3390/biom11101451

**Published:** 2021-10-02

**Authors:** Calvin K. Voong, James A. Goodrich, Jennifer F. Kugel

**Affiliations:** Department of Biochemistry, University of Colorado Boulder, Boulder, CO 80309, USA; calvin.voong@colorado.edu

**Keywords:** HMG protein, HMGB, chromatin, architectural protein, genome organization

## Abstract

High Mobility Group Box (HMGB) proteins are small architectural DNA binding proteins that regulate multiple genomic processes such as DNA damage repair, nucleosome sliding, telomere homeostasis, and transcription. In doing so they control both normal cellular functions and impact a myriad of disease states, including cancers and autoimmune diseases. HMGB proteins bind to DNA and nucleosomes to modulate the local chromatin environment, which facilitates the binding of regulatory protein factors to the genome and modulates higher order chromosomal organization. Numerous studies over the years have characterized the structure and function of interactions between HMGB proteins and DNA, both biochemically and inside cells, providing valuable mechanistic insight as well as evidence these interactions influence pathological processes. This review highlights recent studies supporting the roles of HMGB1 and HMGB2 in global organization of the genome, as well as roles in transcriptional regulation and telomere maintenance via interactions with G-quadruplex structures. Moreover, emerging models for how HMGB proteins function as RNA binding proteins are presented. Nuclear HMGB proteins have broad regulatory potential to impact numerous aspects of cellular metabolism in normal and disease states.

## 1. Introduction

**Figure 1:**
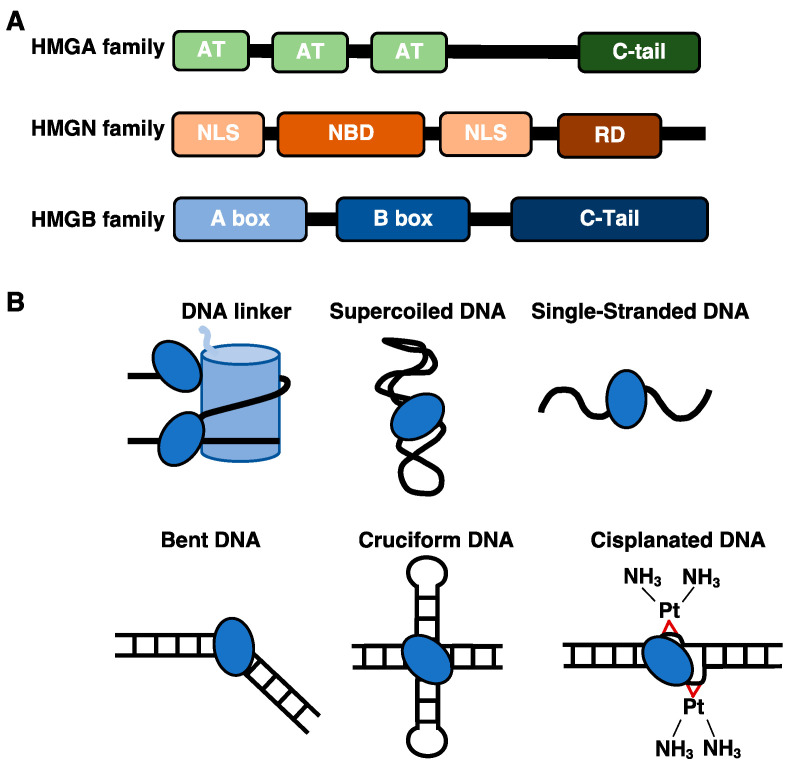
(**A**) Schematic showing the domains in HMG family proteins. Abbreviations: AT, AT hook; C-tail, C-terminal acidic tail; NLS, nuclear localization sequence; NBD, nucleosomal binding domain; RD, regulatory domain; A box, HMG box A; and B box, HMG box B. (**B**) HMGB1/2 can bind to a myriad of DNA structures. The light blue cylinder represents a nucleosome, and the dark blue ovals represent HMGB1/2.

High mobility group (HMG) proteins play essential roles in normal cellular biology by functioning in several intracellular and extracellular capacities. These proteins also contribute to the molecular mechanisms behind the pathology of many disease states, making understanding their regulatory mechanisms particularly important. There are three superfamilies of HMG proteins: HMG AT Hook (HMGA), HMG nucleosome binding (HMGN), and HMG box (HMGB) proteins. The first members of the HMG family were discovered in the 1970s and were initially characterized by their fast migration in an acidic polyacrylamide gel (hence their name) [1]. Within the nucleus, HMG proteins function as architectural DNA binding proteins that dynamically interact with chromatin, therefore modulating DNA-dependent processes. This includes impacting the accessibility of nucleosomal DNA to facilitate protein factor binding, as well as impacting the higher order organization of chromatin structure, therefore regulating DNA replication, DNA repair, transcription, and chromatin remodeling [2]. Each protein within the HMG super family contains one or more HMG domains that define its mechanism of interaction with DNA and/or the nucleosome core particle [3] (Figure 1A). In addition, most HMG proteins contain regulatory domains, typically a C-terminal acidic tail, that can control interactions with the genome or other proteins. After briefly introducing the HMGA and HMGN families of proteins, this review focuses on the HMGB proteins and recent work describing their roles in organizing higher order chromatin structure genome-wide and in binding specific DNA structures and cellular RNAs, ultimately contributing to the regulation of genomic processes in specific disease states. 

### 1.1. The DNA and Nucleosome Binding Properties of HMGA and HMGN Proteins 

The HMGA protein family consists of HMGA1, its splicing variants HMGA1a, HMGA1b, HMG1c, and HMGA2, which are encoded by the HMGA1 and HMGA2 genes respectively [4]. The HMGA proteins interact with DNA and nucleosomes through their unstructured AT hook domains (Figure 1A), which become structured upon binding to DNA [5,6]. HMGA proteins preferentially bind to the minor groove of DNA containing AT-rich stretches [7,8]. They mediate transcriptional activation by promoting the decompaction of chromatin by binding the linker DNA between histones in competition with histone H1 [9,10]. HMGA proteins also coordinate with other members of the HMG family to modulate the compaction and higher order architecture of chromatin, therefore impacting several biological processes that occur on the genome [4,6]. 

The HMGN protein family consists of five members: HMGN1, HMGN2, HMGN3, HMGN4, and HMGN5. These proteins contain a conserved nucleosomal binding domain, a nuclear localization signal, and a C-terminal regulatory domain (Figure 1A). The HMGN proteins directly bind nucleosome core particles via a conserved eight amino acid sequence in the nucleosome binding domain that interacts with an acidic patch on the H2A/H2B dimer, in conjunction with DNA interactions that occur near the entry/exit point of the nucleosome core [11]. HMGN proteins do not displace H1, but instead bind simultaneously [12]. In addition, HMGN proteins can promote the post-translational modifications of the core histones by enhancing the activity of histone modifiers [13]. This helps de-compact chromatin structure and affects the expression of genes and other biological processes that occur on the genome.

### 1.2. The HMGB Proteins Bind Diverse DNA Structures and Interact with Histone Proteins

There are four members in the HMGB family: HMGB1, HMGB2, HMGB3, and HMGB4. These proteins are characterized by their domains that bind to DNA in a structure-specific manner [14] (Figure 1A). Within the nucleus HMGB proteins are highly abundant and ubiquitously expressed; there are approximately 10^6^ molecules of HMGB1, averaging ~1 molecule per 10–15 nucleosomes [14,15]. HMGB proteins function as architectural DNA binding proteins by modulating the local environment of the chromatin to facilitate the binding of regulatory proteins that are important for genomic processes such as transcription and DNA repair. Mammalian HMGB proteins contain two HMG box domains and an acidic C-terminal tail (Figure 1A), except for HMGB4, which lacks the acidic C-terminal tail [16]. HMGB proteins can be post-translationally modified to influence their interactions with DNA, protein partners, and cellular localization. For example, HMGB1 can undergo acetylation, phosphorylation, methylation, ribosylation, and oxidation, creating a complex repertoire of potential regulatory mechanisms [17,18].

The structure of each HMG box consists of three-alpha helices folded into an L-shaped conformation [19,20,21]. Each individual HMG box can bind to DNA in a manner that is not sequence specific but is sensitive to the structure of the DNA [14]. The proteins show an increased affinity for non-B form distorted DNA structures (illustrated in Figure 1B), such as cisplatinated DNA [22], cruciform DNA [23], single stranded DNA [24], supercoiled DNA [25], hemi catenated DNA [26], and DNA mini circles [27]. Moreover, atypical DNA structures can form in cells and play a role in regulating genomic processes through interactions with architectural and regulatory proteins [28,29]. When the HMG boxes bind double stranded DNA, they induce the DNA to bend via specific amino acids that intercalate into the double helix and induce deformation [30]. The acidic C-terminal tails on HMGB proteins are unstructured and consist of stretches of glutamate and aspartate amino acid residues. The acidic C-terminal tail of HMGB1 makes extensive intramolecular interactions with its two DNA binding domains, which dampen its ability to bind and bend DNA [31,32,33,34,35,36,37], impacting the ability of HMGB1 to regulate transcriptional activity [38,39]. 

In the context of chromatin, HMGB proteins can make specific interactions with both the DNA and histone proteins. HMGB proteins have been shown to bind to the linker DNA near the entry/exit junction of the nucleosome [40]. The C-terminal tail of HMGB1 can bind to histone H1, facilitating displacement of the linker histone, allowing HMGB1 to bind the linker DNA to induce nucleosome remodeling and organization of the chromatin [9,41,42,43]. Furthermore, studies have shown that HMGB1 interacts with the histone H3 N-terminal tail, which could facilitate its binding to chromatin [38,44]. It has been proposed that when HMGB proteins bind chromatin they locally modify the structure by bending the DNA and facilitating the binding of regulatory proteins such as transcription factors, chromatin remodelers, and DNA damage repair machinery [14,45,46]. For example, data suggest that HMGB1 facilitates binding of p53 [47] and estrogen receptor [48], among other transcription factors [49]. 

As a function of their interaction with chromatin, HMGB proteins control several genomic processes in response to specific biological cues; therefore, they become essential regulators of cellular programs, for example senescence [50,51,52,53,54], as well as disease states, including cancer, autoimmune diseases, and inflammation [55]. Interestingly, HMGB1, in addition to its roles on the genome, has critical roles in both the cytoplasm and extracellularly. Within the cytoplasm, HMGB1 plays a role in the induction of autophagy, in mitochondrial quality control, and in mitochondrial DNA repair [56,57,58]. The secretion of HMGB1 into the extracellular matrix has been widely studied, where HMGB1 can act as a pro-inflammatory cytokine molecule by binding to cell surface receptors, such as the receptors for advanced glycation end products (RAGE) and toll-like receptor 4 (TLR4), which stimulate the pro-inflammatory response [18]. 

The focus of this review is on the roles of HMGB proteins in the nucleus, emphasizing recent studies that demonstrate how HMGB interactions with the genome control a diversity of cellular programs. We also discuss recent evidence characterizing HMGB protein interactions with G-quadruplex structures that form in promoters and at telomeres, as well as a growing body of data showing that different types of cellular RNA could be important regulatory partners for HMGB proteins. Although the focus of this review is mammalian HMGB proteins, it is worth noting that Saccharomyces cerevisiae Nhp6, a yeast counterpart to HMGB1/2, has been extensively investigated using structural, biochemical, and biophysical approaches, which provide important insight into how HMG boxes interact with DNA and chromatin [59,60,61,62].

## 2. HMGB Proteins Regulate Genome Organization

An important aspect of regulating gene expression is controlling the higher order three-dimensional organization of the genome, such as inter- and intra-chromosomal interactions. Architectural proteins play an important role in this process. For example, topologically associating domains (TADs) are large, looped regions of chromatin in which distal areas of chromosomes are brought together with one another to create chromosomal territories within the nucleus that modulate gene transcription [63]. This three-dimensional architecture can be mapped genome-wide using chromosome conformation capture techniques such as Hi-C. Hi-C can be used to identify TAD boundaries, which are often demarcated by the binding of the architectural protein CTCF (CCCTC-binding factor) and the protein complex cohesion [63]. In addition to higher order genome organization, gene expression is controlled by regulating the compaction of chromatin [64]. Highly compacted heterochromatin silences gene expression and is marked by specific repressive histone modifications such as H3K9 methylation, while euchromatin is considered less compacted and contains more activating histone modifications such as acetylation and H3K4 methylation [65]. Only recently have genome-wide views of HMGB protein occupancy been revealed, providing new insight into how these proteins control both higher order genome organization and chromatin structure.

### 2.1. Regulation of Chromatin Organization and Structure during Senescence

**Figure 2:**
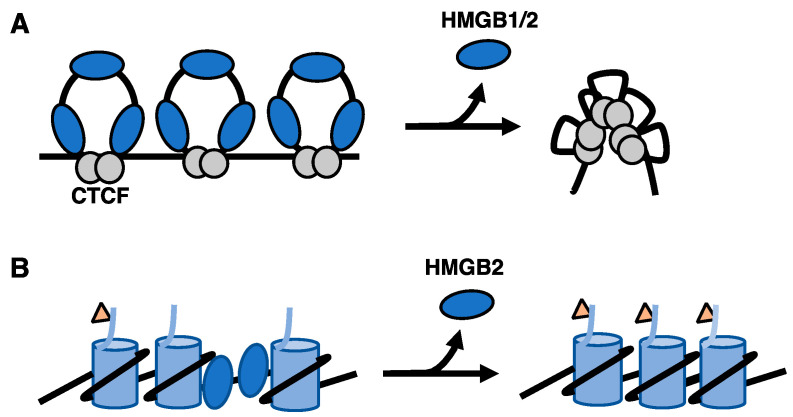
(**A**) HMGB1/2 serves to insulate the interactions between CTCF proteins and demarcate the boundaries of TADs. Loss of HMGB1/2 upon entry into senescence causes clustering of CTCF proteins. (**B**) Loss of HMGB2 during oncogene induced senescence promotes the spread of heterochromatic marks (triangles) into SASP gene loci.

Cellular senescence is a stable arrest of the proliferative state that can be induced by a variety of internal and external stresses, such as prolonged DNA damage, shortening the telomere ends, and oncogene activation [66]. One signature of cellular senescence is the alteration of chromatin structure. HMGB2 is important during replicative senescence. Recent work has shown that HMGB2 is depleted from the nucleus upon entry into cellular senescence, resulting in reorganization of the genome and changes in transcriptional activity [51]. Specifically, using a combination of chromosome conformation capture (Hi-C assays) and ChIP-seq, it was found that that HMGB2 functions to modulate the global chromatin structure and expression of genes found within topologically associating domains (TADs) by insulating against the clustering of CTCF proteins (Figure 2A). Upon senescence entry when HMGB2 was exported from the nucleus, loss of HMGB2 resulted in the clustering of CTCF molecules, as seen in fluorescence imaging, and the compaction of chromatin [51]. The reorganization of TADs was further validated by ChIP-seq data that showed HMGB2 bound to looped TAD regions prior to senescence, helping demarcate the boundaries of sub-TAD regions. HMGB2 was bound to genes involved in senescence processes, such as extracellular matrix reorganization and cellular aging [51]. Upon senescence and the loss of genome-bound HMGB2, the change in genome architecture led to changes in heterochromatin formation and ultimately gene expression. This included an increase in HP1α, a heterochromatic binding protein, and changes in core histone markers, such as a decrease in H3K27me3, consistent with a transition from facultative to constitutive heterochromatin upon senescence entry [51]. 

Recent work shows that HMGB1 also interacts with specific regions of the genome in proliferating cells and is depleted from the nucleus during entry into senescence [50]. Moreover, HMGB1 can be secreted into the extracellular matrix and induce senescence of nearby cells through paracrine signaling, thus playing a dual role in regulating senescence [50]. Within the nucleus, ChIP-seq of HMGB1 showed that the level of chromatin bound HMGB1 decreased upon entry into senescence [50]. Combining these data with Hi-C data revealed that in proliferative cells HMGB1 bound to TAD looped regions and marked a subset of senescence-associated TAD boundaries. Moreover, the HMGB1 clusters were bound to senescence-associated genes [50]. Upon cellular senescence, HMGB1 clusters were disrupted, and the TADs collapsed to form larger domains, similar to what was observed with HMGB2 [51]. This reorganization of the TAD domains as a function of HMGB1 loss during cellular senescence drives the expression of senescence-associated genes and the downregulation of proliferation associated genes. 

During oncogene induced senescence (OIS), which can be caused by mutations in oncogenes such as RAS, BRAF, AKT, E2F1, and cyclin E [66], chromosome condensation occurs to form senescence-associated heterochromatic foci (SAHF), which can be detected as bright punctum in DAPI (4′-6′-Diamidino-2-phenylindole) cell staining [67]. The formation of SAHF has been found to proceed the assembly of HP1 proteins, macroH2A, and H3K9Me onto chromosomes [68], which are all markers of heterochromatin and gene silencing. The binding of HMGB proteins to chromatin during OIS plays an important role in modulating the structure of chromatin during the formation of SAHF. During OIS HMGB2 modulates the expression of senescence-associated secretory phenotype (SASP) protein factors through regulating the chromatin structure of these genes. SASP involves the expression and secretion of cytokines, chemokines, growth factors, metalloproteases, and lipids by senescing cells, which can influence immune responses via paracrine signaling [66]. Using chromatin immunoprecipitation followed by sequencing (ChIP-seq), HMGB2 was found bound to SASP gene loci to promote the expression of SASP factors, such as IL1α, IL8, and IL6 [52]. Knockdown of HMGB2 influenced the global formation of SAHFs by decreasing the percentage of identifiable heterochromatic foci and increasing the spread of heterochromatic marks into the SASP gene loci, therefore silencing the expression of SASP factors and further compacting the genome [52] (Figure 2B). Thus, HMGB2 promotes SASP gene expression by keeping SASP gene loci from being silenced by heterochromatin.

### 2.2. Regulation of the Cardiac Genome by HMGB2

A relationship between HMGB2 and cardiovascular disease was revealed through mass spectrometry experiments that identified chromatin-associated proteins in the hearts of mice in various stages of heart disease [69]. Changes in the chromatin proteome were consistent with global changes to chromatin structure and accessibility. For example, the reprogramming of post-translational modifications on histones and changes in the levels of architectural proteins that control chromatin structure, including HMGB2, were observed. Knockdown of HMGB2 in cardiac myocytes changed gene expression programs related to cell growth and cardiac function and changed overall levels of histone modifications, which suggested a shift toward euchromatin [69]. Further investigation of the molecular role of HMGB2 in orchestrating chromatin organization and gene expression revealed locations across the genome in cardiac cells that were bound by HMGB2 [70]. A comparison with the genomic occupancy of CTCF showed that HMGB2 and CTCF share gene targets; however, these proteins did not bind genes simultaneously in ChIP re-ChIP experiments and they did not colocalize in super-resolution imaging of myocyte nuclei. This leads to a model whereby there is a reciprocal relationship between these two architectural proteins in regulating chromatin organization of the cardiac genome [70]. This in turn helps define the changes in gene expression that occur during the development of cardiac disease. 

### 2.3. Regulation of Genome Organization in a Human Malaria Parasite

A recent study has highlighted a conserved role for HMGB proteins in organizing the genome of a parasite during infection. HMGB1 from the malaria parasite Plasmodium falciparum (pfHMGB1) is important for organizing the higher order conformation of the parasite genome [71]. During infection, pathogenesis involves the transcription of a family of variant malaria genes that express different surface antigens to help escape host immune responses [72]. Expression of family members is mutually exclusive; thus, each malaria parasite only expresses a single variant gene at a given time. This differential expression is controlled by the architectural organization of the variant genes and the localization of individual genes within centromeric or telomeric chromatin domains [73,74,75]. Recent experiments showed that pfHMGB1 is critical for maintaining the centromere/telomere genome organization that controls expression of the variant genes that are critical for pathogenesis [71]. pfHMGB1 was found localized to the centromeres of all 14 P. Falciparum chromosomes by ChIP-seq, along with the centromere-specific H3 variant CenH3 [71]. Upon knockout of pfHMGB1, the structural composition of the centromeres became destabilized, decreasing the intermolecular interactions between centromeres. The disrupted nuclear organization was restored by complementation with the pfHMGB1 gene. Moreover, the HMGB1-dependent changes in genome organization correlated with HMGB1-dependent changes in expression of variant genes. pfHMGB1 knockout down-regulated expression of variant genes, which could also be rescued by complementation. Therefore, in the context of the malaria genome, pfHMGB1 is a critical regulator of genomic architecture, which in turn impacts the pathogenesis of malaria infection. 

## 3. HMGB Proteins Bind G-Quadruplexes with Potential Effects on Cancer

**Figure 3:**
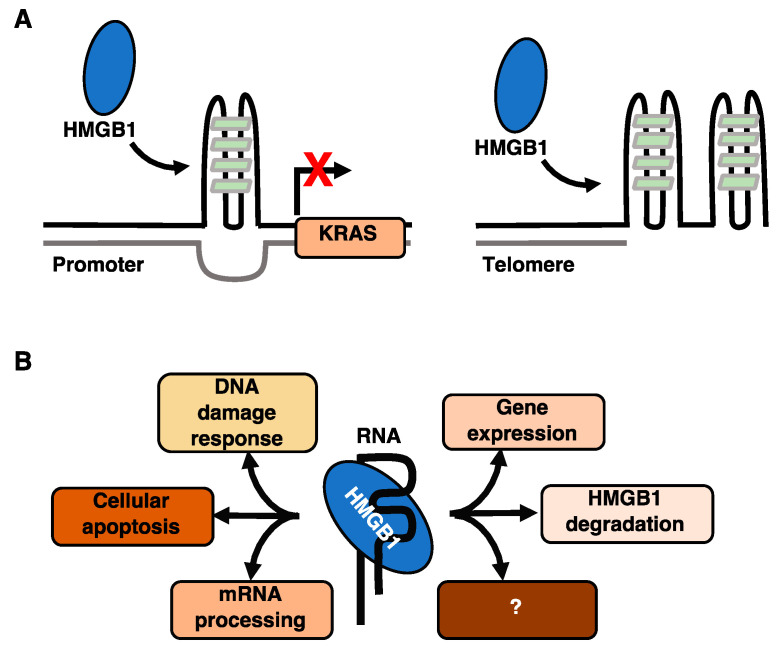
(**A**) HMGB1 binding to the KRAS and telomeric G4-quadruplexes. (**B**) HMGB1/RNA interactions can impact multiple cellular processes.

HMGB1 and HMGB2 are capable of binding to non-B form DNA structures with high affinity, such as those found in cisplatinated DNA, cruciform DNA, and bent-DNA (see Figure 1B). More recently, HMGB1 has been shown to bind G-quadruplex (G4) DNA structures, and in doing so could influence the activity of the enzyme telomerase [76,77] and transcription of the KRAS oncogene [78] (Figure 3A). G4 structures form through the self-association of guanines to form stacked tetrads and could serve to regulate cellular processes that occur on the genome [79,80]. Within the human genome, there are approximately 700,000 G4 structures experimentally identified by G4-seq [81], which are enriched in transcriptionally regulated regions [82]. G4 structures are often found in oncogene promoters, such as the KRAS oncogene [83] and on the ends of telomeres [84] suggesting the G4 structures are important in cancer progression and telomere maintenance. 

### 3.1. The HMGB1 Protein Binds Telomeric G-Quadruplex DNA and Affects the Activity of Telomerase

The dysregulation of telomerase in tumor cells can affect the progression of tumor growth and cellular immortalization due to disruption of the maintenance and extension of the telomeric ends of chromosomes [85]. Proper maintenance of telomeres requires the catalytic activity of the enzyme telomerase, which contains an RNA template that complements the single stranded telomeric overhangs that form after each successive DNA replication. A role for HMGB proteins in telomere homeostasis is beginning to be uncovered. Knockout of HMGB1 in mouse embryonic fibroblasts resulted in decreased telomerase activity, whereas its overexpression increased telomerase activity [76]. Further studies showed that down-regulation of HMGB1 increased the radiosensitivity of human breast cancer cells by dysregulating telomere homeostasis [86]. However, how HMGB1 affects telomerase activity and whether this requires interaction with telomeric DNA were unclear. Recently, HMGB1 was shown to co-localize with TRF1, a telomeric binding protein, and to localize to telomeres via ChIP [78]. Knockdown of HMGB1 in these cells resulted in an increase in DNA damage at telomere ends as measured by the accumulation of ϒH2AX, a histone variant that is a marker for DNA damage. These data suggest that the binding of HMGB1 at telomeric ends could serve to protect the ends of chromosomes from damage [78,85]. Telomere ends contain guanine-rich DNA repeat sequences (TTAGGG) that have the propensity to fold into G4 structures [87] (Figure 3A, right panel). Recent studies investigated the binding of telomeric G4 structures by HMGB1 using structural and biophysical approaches, characterizing in detail the parameters that govern this interaction [78]. Although HMGB1 binds to the G4 telomeric structures and localizes to the telomere ends, it remains unclear whether HMGB1 can facilitate the assembly of the telomerase machinery onto G4 structures. 

### 3.2. HMGB1 Binds a G-Quadruplex in the Promoter of the KRAS Oncogene and Regulates Its Transcription

The KRAS gene encodes for a small GTPase transducer that is implicated in the RAS/MAPK signaling pathway. KRAS is upregulated in many cancers, and mutations in the KRAS gene can cause expression of aberrant KRAS proteins that promote the transformation of normal cells into cancerous cells by promoting cellular proliferation, survival, and cancer progression [88]. The promoter of the KRAS gene contains a guanine–rich element that folds into a parallel G4 DNA structure [89]. Stabilization of this G4 structure silences KRAS transcription [89,90]. The protein hnRNPA1 has been shown to bind and destabilize the KRAS G4 structure, resulting in transcriptional activation [91]. Recently, biophysical studies found that HMGB1 can bind to the KRAS G-quadruplex structure with high affinity and stabilize the structure [92] (Figure 3A, left panel). Importantly, knockdown of HMGB1 led to an increase in KRAS expression, supporting the model that HMGB1 binding to the KRAS G4 structure in cells results in transcriptional repression of the KRAS gene and decreased expression of the KRAS protein [92]. Given the role of KRAS in cancer, and the propensity of other oncogene promoters to contain G4 structures, this regulatory mechanism involving HMGB1 warrants further investigation. 

## 4. A New Regulatory Role for HMGB Proteins as RNA Binding Factors

HMGB proteins are known to be architectural DNA binding proteins; however, a growing body of evidence shows that HMGB proteins can also function as RNA binding proteins. Although the biological roles and diversity of RNA targets remain to be revealed, data suggest that HMGB/RNA complexes have the potential to play important regulatory roles in the cell. Early evidence of HMG proteins binding to RNA was provided by the discovery that the drosophila HMG protein (HMG-D) binds to the minor groove of the transactivation response region (TAR) A-form RNA from HIV-1 and to the rev binding protein element RNA [93]. Other studies found that recombinant rat HMGB1 bound to the long, branched *E. Coli* 5S rRNA and the *Azoarcus* ribozyme in vitro with high affinity, and in the latter case HMGB1 influenced RNA splicing activity [94]. More recently HMG box proteins were found in proteomics screens aimed at comprehensively identifying RNA binding domains in human cells [95,96]. As studies of interactions between specific RNAs and HMGB proteins grow, a clearer view of this regulatory interaction is likely to be revealed (Figure 3B).

### 4.1. HMGB1 Coordinates RNA Metabolism during Senescence Entry

sCLIP (simplified Cross-Linking and Immuno-Precipitation), a high-throughput sequencing method used to identify RNA-protein interactions after crosslinking [97], was used to identify RNA binding partners for HMGB1 in proliferating IMR90 cells [50]. This resulted in identification of non-coding RNAs and senescence-related mRNAs, as well as mRNAs that encode regulators of splicing and chromatin reorganization [50]. Knockdown of HMGB1 altered the splicing of many of its target mRNAs, a portion of which also showed changes in splicing during senescence, suggesting a functional role for HMGB1 in mRNA processing during cellular senescence. In addition, identifying the HMGB1 interactome revealed a myriad of RNA binding proteins (RBPs) that are regulated during senescence, and the mRNAs of some of the RBPs were also bound by HMGB1 [50]. It is possible that a regulatory circuit involving HMGB1/mRNA/RBP interactions participates in regulating senescence and mRNA splicing. Interestingly, studies of the HMGB1 interactome in prostrate epithelial cells identified, among other proteins, members of the SR protein family (SRSFs) [98], which are involved in the regulation of RNA splicing, and were also identified as HMGB1 partners in IMR90 cells during senescence [50]. Other studies have shown a relationship between HMGB1 and SRSF3 in controlling the expression and secretion of the IL-1β mRNA [99], a proinflammatory cytokine that plays a role in SASP activation [100,101]. Further investigations probing how HMGB1 interactions with RNA binding proteins and mRNAs modulate gene expression will likely provide novel insight into new regulatory mechanisms in diverse cell types and diseases.

### 4.2. HMGB1 Interacts with Long Noncoding RNAs to Control Disease States

Long non-coding RNAs (lncRNAs) have been implicated to have roles in a diversity of cellular processes and diseases [102]. It was recently reported that HMGB1 associates with the brain specific DNA damage related lncRNA1 (BS-DRL1) [103]. Data suggest that binding of HMGB1 to BS-DRL1 in neuronal cells directs HMGB1 to sites of DNA damage on chromatin to help facilitate the repair process. Knockdown of BS-DRL1 resulted in decreased HMGB1 binding to chromatin and an increase in DNA breaks, but decreased DNA damage response signaling [103]. Association of HMGB1 with BS-DRL1 is mediated through the N-terminus of the protein, and disruption of this interaction impacted the integrity of the genome by increasing the accumulation of DNA damage, motor dysfunction, and neurodegeneration. Together these results provide compelling evidence that an interaction with a non-coding RNA controls the ability of HMGB1 to bind chromatin in response to DNA damage in neurons. 

In a different biological system, the interaction of HMGB1 with an lncRNA was found to reduce degradation of HMGB1 within the cell and promote tumor cell growth [104]. In multiple myeloma cells, HMGB1 was found to associate with the lncRNA MALAT-1 in a pull-down assay. Interestingly, knockdown of MALAT1 increased the degradation of HMGB1, and increased its ubiquitination, which suggested a potential mechanism for degradation. Treatment with siRNA against MALAT-1 increased apoptosis of the multiple myeloma tumor cells, which was attenuated by expression of HMGB1 [104]. This regulatory network provides new insight into the pathological process of multiple myeloma. It will be interesting to learn of other interactions between HMGB1 and lncRNAs and the mechanisms by which these interactions influence multiple diseases.

## 5. Conclusions and Future Directions

To summarize, HMGB proteins are the most abundant non-histone chromatin binding proteins in the nuclei of mammalian cells. The HMGB proteins are well known for modulating the local chromatin environment, facilitating the binding of other proteins to chromatin, and controlling nuclear processes such as transcription, DNA damage repair, and nucleosome sliding. Here we discussed recent evidence that the role of HMGB proteins in the nucleus extends to regulating global chromatin architecture, telomere maintenance, cellular senescence, and RNA biology. For example, the loss of HMGB1/2 from the nucleus results in the reorganization of genome architecture, which has direct implications for gene expression and ultimately the progression of cancers [51,52], cardiovascular diseases [70,105], and parasitic immune evasion [71]. The binding of HMGB1 to G4 quadruplex DNA stabilizes the DNA structure; at the *KRAS* oncogene this inhibited expression and at telomere ends this protected the DNA from damage [78,92]. Newly identified interactions between HMGB1 and various mRNAs or lncRNAs modulate splicing, gene expression, and the formation of ribonucleoprotein complexes within the cell [50,103,104]. The varying roles of nuclear HMGB1/2 proteins within different cell types highlights the complexity of this family of proteins in regulating genomic processes.

Much remains to be learned about the regulatory roles of nuclear HMGB proteins. It will be interesting to unravel the relationship between genome organization and the HMGB-facilitated binding of transcription factors. This could involve interplay with the formation of local DNA structures such as G4 quadruplexes or cruciform DNA. For example, both p53 and HMGB1 exhibit enhanced binding to cruciform DNA, suggesting that the local DNA structure may play a regulatory role in transcription factor binding facilitated by HMGB proteins [28]. With over 700,000 experimentally identified G4-quadruplex structures in the human genome [81], this is an intriguing possibility. Future studies are required to unravel the relationship between the genomic positioning of local DNA structures, HMGB1/2 binding, localization of specific transcription factors, and the presence of looped regions of chromatin. In addition to revealing how HMGB proteins function on the genome, it will be important to identify the breadth of potential RNA targets of HMGB proteins. Only then will we realize how widespread RNA-mediated regulatory mechanisms for HMGB proteins might be. Lastly, characterizing how these regulatory mechanisms differ in healthy versus diseased cells is critical for future understanding of how HMGB proteins contribute to disease etiology and progression. 

Studies have suggested that HMGB1 has the potential to be a therapeutic target, as reviewed in detail elsewhere [106]. In addition, it is possible nuclear HMGB1 could serve as a prognostic biomarker, given its elevated levels in cancer cells that reflect the proliferative state of the cell [107]. The majority of studies of nuclear HMGB proteins have been performed in cells and in vitro. In the future, such studies could extend to in vivo models. Indeed, a conditional knockout mouse for HMGB1 has been developed [108], which will facilitate such efforts. Future work will undoubtedly reveal the potential of HMGB proteins to serve as therapeutic and/or diagnostic molecules. 

## Data Availability

Not applicable.
